# Fluoroquinolone-Resistant *Campylobacter* Isolates from Conventional and Antibiotic-Free Chicken Products

**DOI:** 10.1289/ehp.7647

**Published:** 2005-02-02

**Authors:** Lance B. Price, Elizabeth Johnson, Rocio Vailes, Ellen Silbergeld

**Affiliations:** Johns Hopkins University, Bloomberg School of Public Health, Baltimore, Maryland, USA

**Keywords:** bacterial, *Campylobacter*, chickens, drug resistance, drugs, fluoroquinolones, food microbiology, methods, poultry, veterinary

## Abstract

The use of fluoroquinolones (FQs) in poultry production is an important issue in public health today. In February 2002, two prominent U.S. poultry companies pledged to stop using FQs for flock-wide treatment. One year later, we began a survey of *Campylobacter* isolates on chicken products from these two companies and from two producers claiming total abstention from antibiotic use. Using both standard isolation methods and new methods modified to enhance detection of FQ-resistant *Campylobacter*, we compared rates of FQ-resistant *Campylobacter* among these products. Four major findings were drawn from this study: *a*) antibiotic-free brands were not more likely to be contaminated with *Campylobacter*; *b*) a high percentage of products from the two conventional brands were contaminated with FQ-resistant *Campylobacter* (43 and 96%); *c*) these conventional brands had significantly higher odds of carrying resistant strains compared with antibiotic-free products; and *d*) supplementing media with FQs increased the sensitivity of detecting FQ-resistant strains among mixed populations of *Campylobacter*, thus reducing a bias toward underestimating the prevalence of FQ-resistant *Campylobacter* on samples. These results suggest that FQ resistance may persist in the commercial poultry environment in the absence of FQ-selective pressure and that these strains contaminate a larger proportion of foods than reported previously.

Microbiologic and epidemiologic investigations have begun to elucidate the major sources of fluoroquinolone (FQ)-resistant *Campylobacter* infections in the United States. Major findings include the following: *a*) an increasing proportion of isolates collected in the United States from human *Campylobacter* infections are resistant to FQs [Allos 2001; Centers for Disease Control and Prevention (CDC) 2003; Nachamkin et al. 2002]; *b*) studies of human stool samples taken before FQ therapy indicate that most of the infections were resistant before treatment (Gaunt and Piddock 1996; Smith et al. 1999); *c*) epidemiologic studies indicate that fresh poultry products are the major sources of *Campylobacter* infections in humans (Harris et al. 1986; Neimann et al. 2003); *d*) FQ-resistant *Campylobacter* populations develop quickly in *Campylobacter*-infected chickens that are treated with FQs (McDermott et al. 2002); and *e*) in the United States, consumer-ready fresh poultry products are commonly contaminated with FQ-resistant strains of *Campylobacter* (Ge et al. 2003). It has been inferred from these findings that a large number of FQ-resistant *Campylobacter* infections in humans are the result of FQ use in the poultry house. Some of the strongest evidence to support this connection comes from a recent study of human campylobacteriosis in Australia, where despite regular clinical use of FQs and normal rates of *Campylobacter* infection, there are no confirmed cases of domestically acquired FQ-resistant campylobacteriosis (Unicomb et al. 2003). The authors concluded that this dramatic phenomenon is likely related to Australia’s prohibition of FQ use in poultry production.

FQs, Sara Flox WSP (sarafloxacin water-soluble powder; Abbott Laboratories, North Chicago, IL) and Baytril (enrofloxacin; Bayer Corporation, Shawnee Mission, KS), have been approved for use in the United States to control *Escherichia coli* infections in broiler chickens since 1995 and 1996, respectively [Food and Drug Administration (FDA) 1995, 1996]. In October 2000, the FDA’s Center for Veterinary Medicine announced that it intended to withdraw approval for FQs in poultry production because new evidence had shown that it may not be safe for human health (FDA 2000a). In March 2004, the FDA’s regulation was upheld in court, but Bayer has appealed this decision. Although this battle continues, Baytril continues to be marketed for use as a drinking water additive for flock-wide application in broiler production. The exact number of birds treated with Baytril annually is not publicly available, but Bayer estimated that < 1% of the American poultry flock was treated with the drug in 2001 (Bayer Healthcare 2004). Given the enormous scale of the American poultry flock (~ 8.4 billion in 2001), this estimate indicates that up to 84 million birds may be treated with Baytril annually [U.S. Department of Agriculture (USDA) 2002]. In February 2002, in response to public concerns regarding FQ use in poultry production, two major U.S. producers, Tyson and Perdue, separately announced that they would stop using FQs in drinking water to treat poultry flocks (Perdue Farms 2002; Tyson Foods 2002).

A recent survey of *Campylobacter* from raw poultry products indicated that 35% of isolates were resistant to ciprofloxacin (CIP) (Ge et al. 2003). Ge et al. (2003) used standard FDA methodologies in which *Campylobacter* colonies were isolated on *Campylobacter* media and a small sample of colonies (typically no more than three) was tested for susceptibility to FQs. Because poultry products are often contaminated with mixed populations of *Campylobacter* strains with differing FQ susceptibilities, this assay has questionable sensitivity, and the 35% figure reported previously is considered to underestimate the actual prevalence of FQ-resistant strains (FDA 2000b).

In the present study, we performed a survey of fresh poultry products from two of the countries largest conventional producers, Tyson Foods and Perdue Farms, and two “antibiotic-free” producers, Bell & Evans and Eberly. *Campylobacter* strains were isolated using standard FDA methodology and also by a modified method that included FQ-supplemented agar medium to identify resistant strains among a mix of susceptible and resistant strains. We analyzed our results comparing FQ-resistant *Campylobacter* carriage among the two “antibiotic-free” and the two conventional brands and tested the increased sensitivity gained by using FQ-supplemented agar.

## Materials and Methods

### Sampling and enrichment.

Fresh chicken products from two antibiotic-free producers, Bell & Evans (Fredericksburg, PA; A) and Eberly Poultry (Stevens, PA; B), and two conventional producers, Perdue Farms (Salisbury, MD; C), and Tyson Foods (Springdale, AR; D), were purchased seven to eight times from grocery stores in the Baltimore, Maryland, area over the course of 10 weeks (from 25 February to 13 May 2003; Table 1). All samples were purchased in packaging applied at the processing plant, and all products were “bone-in” and “skin-on” (i.e., not skinless boneless products). On most occasions, three separate packages from the four producers were purchased each time; all three samples were purchased from the same grocer on a given date and were typically from the same production lot. Packages were refrigerated at 4°C until they were sampled (within 48 hr of purchase).

A single piece of chicken was sampled from each package. The package was wiped with 70% ethanol and cut open with a new disposable razor blade, and the plastic cover was removed and photocopied for record keeping. Sterile forceps were used to transfer a single piece of chicken to a stomacher bag containing 200 mL sterile Bolton broth supplemented with laked horse blood (Oxoid, Ogdensburg, NY; Quad Five, Ryegate, MT). Samples were then shaken by hand for 2 min. Chicken was removed using forceps, and the bag was sealed 1–2 cm above the top of the broth. Enrichments were incubated at 42°C for 22–26 hr.

### Isolation.

Ten microliters of the enrichment [~ 10^6^ colony forming units (CFU)] was streaked onto Abeyta-Hunt agar (Hunt et al. 2000) with and without 4 μg/mL CIP (U.S. Biological, Swampscott, MA) and incubated for 22–26 hr at 42°C. A single typical *Campylobacter* colony from each of the two media was streaked for isolated colonies on *Campylobacter* blood agar (Fisher Scientific, Hampton, NH). A single purified colony was then streaked for confluent growth on *Campylobacter* blood agar and incubated for 22–26 hr. A 10-μL loopful of cellular material was transferred to *Campylobacter* freezing medium (Hunt et al. 2000).

### DNA isolation.

DNA was isolated from a second loopful of material using the DNeasy tissue kit (Qiagen, Valencia, CA).

### Species confirmation.

Presumptive *Campylobacter* isolates were confirmed and the species identified using a polymerase chain reaction (PCR) amplification/restriction digest protocol described previously (Engvall et al. 2002). Briefly, THERM1 and THERM4 PCR primers were used to amplify a region of DNA specific to thermophilic members of the genus *Campylobacter*. This PCR product was then digested in two separate reactions using the restriction endonucleases *Alu*I and *Tsp*509I. The species-specific restriction patterns produced from this digestion enabled us to identify the species of each isolate.

### Susceptibility.

Susceptibility to CIP was determined using standard Clinical and Laboratory Standards Institute and *Campylobacter*-specific methods described previously (McDermott and Walker 2003). Briefly, *Campylobacter* isolates were grown overnight on *Campylobacter* blood agar (Fisher) under microaerophilic conditions. Colonies were suspended to approximately 0.5 McFarland standard in Mueller-Hinton broth and inoculated onto Mueller-Hinton agar supplemented with 5% sheep blood and CIP (U.S. Biological) ranging from 0.12 to 32 μg/mL. Plates were grown 22–26 hr at 42°C under microaerophilic conditions. The reference strain used was *Campylobacter jejuni* ATCC 33560 (American Type Culture Collection, Rockville, MD). Strains were designated resistant if their minimum inhibitory concentration was > 4 μg/mL.

### gyrA QRDR sequence analysis.

The nucleotide sequence of the quinolone-resistance–determining region (QRDR) of *gyrA* was sequenced from isolates using the following primer pair designed from the *Campylobacter* whole-genome DNA sequence (Parkhill et al. 2000): Cj gyrA QRDR F, GCC TGA CGC AAG AGA TGG TTT A; and Cj gyrA QRDR R, TAT GAG GCG GGA TGT TTG TCG. Multilocus sequence typing analysis was used to further characterize some isolates as described previously (Dingle et al. 2001).

### Statistical analysis.

We performed statistical analyses using Stata 8.0 (StataCorp, College Station, TX). We used Fisher’s exact tests to compare the rates of undifferentiated *Campylobacter* (susceptible and resistant) carriage and FQ-resistant *Campylobacter* carriage across the brands. Odds ratios (ORs) of undifferentiated and FQ-resistant *Campylobacter* carriage with corresponding exact 95% confidence intervals (CIs) were computed for all pair-wise comparisons across the brands.

## Results

Overall, *Campylobacter* was detected on 84% of the chicken tested, and FQ-resistant strains were detected on 17% using unsupplemented media and on 40% using agar supplemented with 4 μg/mL CIP (Table 2). When the two methods resulted in the isolation of strain pairs of different FQ susceptibilities, analysis of the *gyr*A, *asp*A, *gln*A, *glt*A, *gly*A, *pgm*, and *tkt* genes from the two isolates revealed that most of these pairs (19 of 21) also differed at two or more nucleotides (data not shown). That is to say, 19 of the 21 resistant isolates were shown to be genetically unique from their susceptible counterparts and would have been missed using standard isolation techniques (i.e., unsupplemented media). In contrast, only two strains were found to be within a single polymorphism (among the seven genes examined) of their susceptible counterparts. Therefore, as potential sporadic mutants isolated by chance, these two strains represent the maximum potential loss in assay specificity.

DNA sequence analysis of the *gyr*A gene revealed that all resistant strains isolated in this survey had a Thr86→Ile substitution, as reported previously by Wang et al. (1993).

Using Fisher’s exact test, we were able to reject the null hypothesis that the rates of undifferentiated *Campylobacter* (susceptible and resistant) carriage are the same for all the brands (*p* < 0.001). Table 3 displays the pair-wise odds ratios of undifferentiated *Campylobacter* carriage and exact 95% CIs among the brands. We found that brand A (antibiotic-free) had significantly lower carriage rates compared with the other producers.

Given the robustness of the selective assay data, all comparative statistical analyses were performed using the data resulting from the CIP-supplemented media. We found statistically significant differences in the rates of FQ-resistant *Campylobacter* carriage across the brands (Fisher’s exact test, *p* < 0.001). The pairwise analysis of FQ-resistant *Campylobacter* carriage among the brands is presented in Table 4. Brand D (conventional) had a 96% carriage rate for FQ-resistant *Campylobacter* and significantly higher odds of carrying these strains than did each of the other brands. The biggest difference in odds of FQ-resistant *Campylobacter* carriage was observed when comparing brand D with brand B (antibiotic-free). Specifically, we estimated that the odds of FQ-resistant carriage in brand D was 460 times greater than the odds of FQ-resistant carriage in brand B (95% CI, 21.7–19766.8). There was no significant difference between the two antibiotic-free brands when compared with one another.

## Discussion

### Limitations of the study.

The present study has three primary limitations. First, the study was limited in the number of stores sampled and its geographical region. However, this was not likely to reduce the generalizability of the results because we used products that are widely distributed in the United States. Moreover, we carefully selected packages that were sealed at the processing facility by the producer so that store-related factors would not affect the prevalence of FQ-resistant *Campylobacter*. Second, the sampling period (25 February to 13 May 2003) was relatively short. Because of this, we could not detect any potential seasonal variation in carriage rates. Finally, we were not able to test the same cut (e.g., thigh) each time from each producer, which would have been ideal. This was because thighs were not available for brand B during the testing period. However, given that we measured the presence or absence of *Campylobacter* on chicken products, rather than the quantity of *Campylobacter* on a given sample, this variation likely did not affect the overall outcome of the study.

### *Lingering FQ-resistant* Campylobacter.

On 18 February 2002, Tyson announced that it planned to discontinue FQ use and estimated use in previous years to be approximately 0.2% (Tyson Foods 2002). Shortly thereafter, on 25 February 2002, Perdue announced that they would immediately stop using FQ and also claimed FQs had not been used within the previous year (Perdue Farms 2002). However, 1 year later, we found significant proportions of products from both of these companies that carried FQ-resistant strains of *Campylobacter*. Accepting the veracity of these announcements, our data suggest that past FQ use may have persistent effects on *Campylobacter* populations in poultry houses. This is consistent with reports from Denmark indicating that vancomycin-resistant enterococci could be isolated from broiler flocks 5 years after avoparcin was banned for use in broilers in that country (Heuer et al. 2002). These studies challenge the notion that resistant populations will quickly revert to a susceptible state once antimicrobial pressure is removed. Indeed, models indicate that microbes may be more likely to develop compensatory mutations that ease the metabolic costs of resistance determinants rather than simply revert back to a susceptible phenotype (Levin et al. 2000).

### Inadequate hygiene.

If what we observed is an indication of lingering resistance, it may be important to improve cleaning and disinfection between flocks. Biofilms in water distribution systems have been identified as potential sources of *Campylobacter* infection in poultry houses (Trachoo et al. 2002). Baytril is administered through poultry drinking water systems; therefore, this could be an important reservoir of FQ-resistant *Campylobacter*. Studies using molecular fingerprinting techniques have provided mixed indications of the importance of insufficient floor sanitation to the carryover of *Campylobacter* isolates, and it is clear that some strains do persist from flock to flock (Petersen and Wedderkopp 2001; Shreeve et al. 2002). This problem may be magnified in the United States, where many poultry houses are built with dirt floors and are typically cleaned only every 2–3 years (Hayes et al. 2000). This practice may support a long-term reservoir for FQ-resistant *Campylobacter* infections of subsequent flocks.

### Cross-contamination.

The microbes on fresh poultry products may reflect the cecal contents of the individual bird at harvest as well as conditions in the processing plant. Modern plants can process > 200,000 broilers/day, and *Campylobacter* strains found on poultry carcasses after processing can be significantly different from those found in the flock before slaughter (Newell et al. 2001). Therefore, the presence of antimicrobial-resistant *Campylobacter* on a particular broiler carcass may result from contamination of the slaughter equipment by a broiler flock processed previously. This issue is particularly relevant to the antibiotic-free producers whose broiler products may become contaminated with antimicrobial-resistant bacteria in abattoirs that process both antibiotic-free and conventional flocks. Both of the antibiotic-free producers included in this study process their broilers in facilities that are also used for antimicrobial-treated flocks. However, no FQ-treated flocks are said to be processed in the brand A abattoir (Ranck S, personal communication). Such a claim could not be made for the brand B abattoir (Carson E, personal communication).

### Enhancing the sensitivity of detecting antibiotic resistance in food isolates.

The sensitivity of antibiotic resistance surveys can be significantly enhanced by including a selective step in the isolation procedure. The *Campylobacter* isolation methodology recommended by the FDA and used in the National Antibiotic Resistance Monitoring Service (NARMS) does not include a selective isolation step. Without such a step, this method is likely to underestimate the presence of resistant strains when they exist among a group of mixed susceptibility strains. The results of the present study indicate that the risk of underestimation using nonselective media likely outweighs the risk of potential false positives due to sporadic mutation during enrichment. We observed no difference between the numbers of resistant isolates determined by the two methods on the brand A (antibiotic-free) samples. Furthermore, despite the fact that most brand B (antibiotic-free) samples were positive for *Campylobacter* (20 of 21), only one sample gave conflicting results using the two different assays. DNA sequence analysis revealed that the two strains isolated from this sample were genetically distinct (i.e., differed by more than one polymorphism).

*Campylobacter* strains have been shown to develop quinolone resistance at an average rate of 5 × 10^−9^ (Taylor et al. 1985; Wang et al. 1993; Wang and Taylor 1990), with a few strains reported to develop at rates as high as 5 × 10^−7^ (Bachoual et al. 2001; Payot et al. 2002; Wang et al. 1993). Using the standard FDA protocol, approximately 10^6^ CFU are transferred to a plate from enrichment (assuming a 10-μL calibrated loop and an overnight *Campylobacter* culture grown in Bolton broth). Therefore, on average, one would expect to isolate a random mutant with a frequency of approximately 1 in 200 assays. In the present study, sequence typing analysis was used to examine the genetic background of strains isolated using the two different methods. In 19 of 21 cases, the strains differed at multiple nucleotides unrelated to resistance. The most parsimonious explanation for the observed difference in strains isolated by the two methods is that these poultry samples were contaminated with mixed populations of FQ-susceptible and FQ-resistant strains. Under such conditions, the likelihood of isolating resistant strains using nonselective conditions is dependent upon variables such as relative starting concentration and doubling time of individual strains in the enrichment broth. Using the selective method minimized potential masking of the effects of such variables.

### Implications for public health.

Despite high incidence rates, mortality due to *Campylobacter* infections is rare in the United States. Most healthy individuals are thought to pass infections without the aid of antimicrobial therapy within 7–10 days. In contrast, antimicrobial therapy can be critical for the treatment of *Campylobacter* infections in the elderly and the immunocompromised (Djuretic et al. 1996; Manfredi et al. 1999; Tee and Mijch 1998). CIP is a commonly prescribed antimicrobial for campylobacteriosis (Nachamkin et al. 2002), and the emergence of FQ-resistant *Campylobacter* strains magnifies the threat to at-risk populations (Anderson et al. 2003). Therefore, it is critical to accurately measure the prevalence of FQ-resistant *Campylobacter* in the food supply and to identify the factors contributing to their presence.

## Figures and Tables

**Table 1:** Summary of dates and samples tested.

	Purchase dates							
Brand	25 Feb	4 Mar	11 Mar	25 Mar	1 Apr	22 Apr	29 Apr	13 May
A	T	T	T	T	T	T	T	T
B*^a^*	N	W	W	W	W	D	D	L
C	T	T	T	T	T	N	T	T
D	T	T	T	T	T	T	T	T

Abbreviations: D, drumstick; L, leg; N, no sample; T, thigh; W, whole chicken.

a Thighs from antibiotic-free brand B were not available in the Baltimore area at the time of the sampling.

**Table 2:** Percentage (*n*) of samples testing positive for *Campylobacter* and FQ-resistant *Campylobacter* carriage, by brand and medium.

	Brand (no. of samples)	Undifferentiated *Campylobacter*	Nonselective medium, CIP-resistant *Campylobacter*	Selective medium, CIP-resistant *Campylobacter*
Antibiotic-free	A (24)	54 (13)	13 (3)	13 (3)
	B (21)	95 (20)	0 (0)	5 (1)
Conventional	C (21)	90 (19)	19 (4)	43 (9)
	D (24)	100 (24)	33 (8)	96 (23)
Total	All (90)	84 (76)	17 (15)	40 (36)

**Table 3:** Pair-wise comparisons of the odds of undifferentiated *Campylobacter* (susceptible and resistant) carriage among brands.

Reference brand	Comparison brand	OR*^a^* (95% CI)*^b^*	*p*-Value*^c^*
A	B	16.9 (1.9–763.8)	< 0.01
	C	8.0 (1.3–85.6)	0.01
	D	40.6 (4.8–∞)	< 0.01
B	C	0.5 (0.01–10.0)	> 0.99
	D	2.4 (0.0–∞)	0.47
C	D	5.0 (0.61–∞)	0.21

a Zero counts were replaced with 0.5 in order to estimate ORs for comparisons with D.

b 95%CIs are based on exact methods; upper bounds of the 95% CI were not estimable for comparisons with D.

c Based on Fisher’s exact test for each pair-wise comparison.

**Table 4:** Pair-wise comparisons of the odds of FQ-resistant *Campylobacter* carriage among brands.

Reference brand	Comparison brand	OR (95% CI)	*p*-Value
A	B	0.4 (0.01–4.9)	0.61
	C	5.3 (1.0–34.7)	0.04
	D	161.0 (13.5–6924.0)	< 0.01
B	C	15.0 (1.6–689.3)	< 0.01
	D	460.0 (21.7–19766.8)	< 0.01
C	D	30.7 (3.3–1365.6)	< 0.01
“Antibiotic-free”	Conventional	25.2 (6.8–111.4)	< 0.01
